# Dose-dependent effect of cannabinoid WIN-55,212-2 on myelin repair following a demyelinating insult

**DOI:** 10.1038/s41598-019-57290-1

**Published:** 2020-01-17

**Authors:** J. Tomas-Roig, H. Y. Agbemenyah, N. Celarain, E. Quintana, Ll. Ramió-Torrentà, U. Havemann-Reinecke

**Affiliations:** 1 Department of Psychiatry and Psychotherapy, University of Göttingen and Center Nanoscale Microscopy and Molecular Physiology of the Brain (CNMPB), Göttingen, Germany; 2 Girona Neuroimmunology and Multiple Sclerosis Unit (UNIEMTG), Dr. JosepTrueta University Hospital and Neurodegeneration and Neuroinflammation research group, Girona Biomedical Research Institute (IDIBGI), Girona, Spain; 3 Laboratory for Aging and Cognitive Diseases, European Neuroscience Institute, Göttingen, Germany and University of Health and Allied Sciences, Ho, Ghana

**Keywords:** Proteins, Gene expression, Multiple sclerosis, Animal behaviour

## Abstract

Dysfunctions in the endocannabinoid system have been associated with experimental animal models and multiple sclerosis patients. Interestingly, the endocannabinoid system has been reported to confer neuroprotection against demyelination. The present study aims to assess the effects of the cannabinoid agonist WIN-55,212-2 in cuprizone fed animals on myelin repair capacity. Animals exposed to cuprizone were simultaneously treated withWIN-55,212-2, behaviorally tested and finally the corpus callosum was exhaustively studied by Western blotting, qRT-PCR and a myelin staining procedure. We report that the long-term administration of WIN-55,212-2 reduced the global amount of CB_1_ protein. Histological analysis revealed clear demyelination after being fed cuprizone for three weeks. However, cuprizone-fed mice subjected to 0.5 mg/Kg of WIN-55,212-2 displayed no differences when compared to controls during demyelination, although there was a robust increase in the myelinated axons during the remyelination phase. These animals displayed better performance on contextual fear conditioning which was in turn non-attributable to an antinociceptive effect. In contrast, a 1 mg/Kg dosage caused a remarkable demyelination accompanied by limited potential for myelin repair. Upon drug administration while mice ongoing demyeliniation, the expression of *Aif1* (microglia) and *Gfap* (astrocytes) followed a dose-dependent manner whereas the expression of both markers was apparently attenuated during remyelination. Treatment with vehicle or 0.5 mg/Kg of the drug during demyelination increased the expression of *Pdgfra* (oligodendrocyte precursor cells) but this did not occur when 1 mg/Kg was administered. In conclusion, the drug at 0.5 mg/Kg did not alter myelin architecture while 1 mg/Kg had a deleterious effect in this model.

## Introduction

The destruction of the myelin sheath in the central nervous system (CNS) is prominent in many clinico-pathologic conditions like multiple sclerosis (MS)^1^. MS is a chronic inflammatory disorder of the CNS characterized by inflammation and progressive axonal neurites injury terminating in neurodegeneration^2^. The use of the main active component of marijuana, Δ9-tetrahydrocannabinol (*THC*), has a broad range of therapeutic effects for a variety of medical conditions, including pain, anxiety, glaucoma, and emesis, and also possesses a neuroprotective effect^3–6^. For our research, we chose the cannabinoid agonist WIN-55,212-2, since this chemical compound has been shown to possess better efficacy at CB_1_-receptor (Ki = 1.9 nM) than *THC* (Ki = 41 nM) and shows greater binding affinity to CB_1_ than CB_2_^7,8^. Among animal models that reproduce the clinico-pathological features of MS, the murine model of cuprizone (CPZ) feeding is a simple and reliable model well characterized in C57BL/6 mice strain for inducing and studying de- and remyelination behind non-autoimmune-mediated demyelination^9,10^. T he administration of the neurotoxicant CPZ leads to olig odendrocyte cell death, microgliosis and astrogliosis^10^. The pathophysiology of CPZ has been extensively evaluated under distinct conditions and paradigms^11^.The endocannabinoid system is deregulated in MS (for review see^12^) and also participates in different forms of synaptic plasticity essential for cognitive and emotional behaviors^13–18^ like fear expression^19^.

With the rationale that the endocannabinoid signaling through the cannabinoid receptors confers neuroprotection during acute demyelination^5^ and also participates in distinct phases of conditioned fear^19^, we hypothesized that the use of the cannabinoid agonist WIN-55,212-2 (WIN) in CPZ-fed mice could differentially affect the mice response to fear as well as the myelin repair following a demyelinating insult.

## Methods

A cohort of 130 C57BL/6 male mice at age of 6–7 week was purchased from Charles River Laboratories (Sulzfeld, Germany). Upon arrival, the animals were housed five mice per cage and kept under standard conditions (12 h light/dark cycle with 6:00/18:00 lights on/off, room temperature of 21 ± 2 °C and food and water *ad libitum*). All procedures were approved by the Göttingen University Institutional Animal Care and Use Committee and were in accordance with NIH guidelines for the use of animals in research and the European Communities Council Directive (2010/63/EU).

### The cuprizone murine model

**Figure 1:**
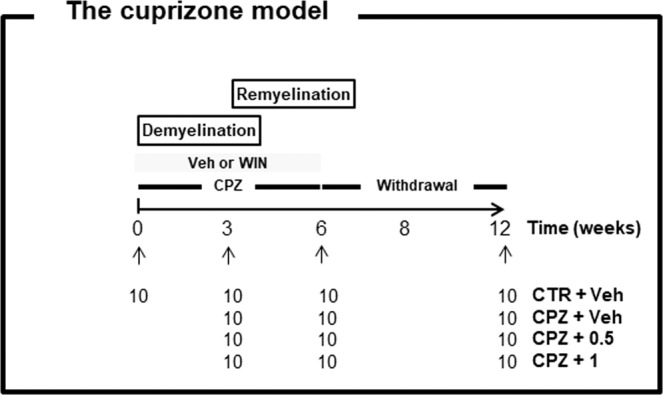
Schematic drawing of the experiment. Mice were divided into a control group fed with a regular chow and a treatment group that received a diet supplemented with 0.2% cuprizone for 3 or 6 weeks. In the recovery group, animals were fed with the CPZ-containing diet for 6 weeks, followed by 6 weeks on a regular diet. At the same time, those animals fed with CPZ were intraperitoneally treated with WIN-55,212-2 (WIN) or phosphate buffered saline (vehicle) once per day. N = 10. Control, animals fed with standard diet and treated with phosphate buffered saline (Veh); CPZ, cuprizone-fed animals; WIN, WIN-55,212-2.

After a one-week period of habituation, the mice were divided into two groups: 1) control animals fed with a standard diet, and 2) treated animals subjected to a diet supplemented with 0.2% CPZ for three or six weeks. In the recovery group, mice were fed with the neurotoxicant for six weeks, followed by six weeks on a regular based diet. At the same time, those animals exposed to CPZ were intraperitoneally treated with WIN (Sigma–Aldrich, Hamburg, Germany) or the vehicle solution (referred to throughout the text as CPZ) composed of 10% DMSO, 0.1% Tween80 in 0.9% phosphate buffered saline (all from Sigma–Aldrich, Hamburg, Germany). The drug WIN was dissolved in an identical vehicle-based solution and prepared freshly every day. The same volume of WIN or the vehicle (200 μl) was i.p. applied once per day (WIN at 0.5 and 1 mg/kg). At predetermined time points (3, 6 and 12 weeks) methylene blue AZUR II staining and quantitative RT-PCR were used to validate both de- and remyelination processes, as has been previously described^10,11^. Figure 1 shows a schematic representation of the experiment.

### Animal testing

The behavioral effects of WIN-55,212-2 in mice were assessed 30 min after the last injection by measuring analgesia^20^ and fear conditioning^21^. A distinct cohort of mice was subjected to the same experimental procedure to evaluate the nociceptive effects of the drug WIN-55,212-2 (n = 15). Analgesia was determined by use of hot plate test. Analgesia was evaluated with a hotplate apparatus (Columbus Instruments, Ohio, USA) heated to 52 °C (−0,1 °C). The latency until rodents displayed first signs of pain (licking or flinching of the hindpaws, jumping) was accurately registered. The cutoff time was set to 60 sec^20^. Associative learning memory was monitored by TSE fear conditioning box (Hamburg, Germany). Mice were exposed to a training protocol which consisted of a single context exposure (3 min) followed by a tone [30 sec, 10 kHz, 75 dB sound pressure level (SPL)] and a foot shock (2 sec, 0.7 mA, constant current). The probe test was done 24 hours later by re-exposing the animals for 3 min into the same context and into novel context for 3 min exposure to a tone (10 kHz, 75 dB SPL). The freezing behavior was automatically registered (n = 10).

### Brain samples collection and tissue evaluation

Once animals were behaviorally tested, they were deeply anesthetized by i.p. injection of 2,2,2-tribromo-ethanol (Sigma-Aldrich, Hamburg, Germany) and then transcardially perfused with 0.1% phosphate buffered saline (PBS). The brains were surgically removed, fixed with 4% paraformaldehyde (PFA) (Serva, Heidelberg, Germany) and postfixed in 2.5% glutaraldehyde (Science Services, Munich, Germany). Finally, the corpus callosum was postfixed with OsO_4_ (Science Services, Munich, Germany) in phosphate buffer pH 7.3 and embedded in EPON resin after dehydration. Serial 35 µm thick coronal sections were cut using a ultramicrotome (Leica, Vienna, Austria) for the staining of myelinated fibers (n = 3). At the same time, the corpus callosum was freshly microdissected under binocular microscope and frozen in liquid nitrogen for Western blotting (n = 4) and quantitative RT-PCR analysis (n = 3).

### Counting of axons in semi-thin sections

The coronal sections (interaural line 1, bregma −2.155 mm) were properly stained with methylene blue AZUR II and observed under light microscope (Olympus light microscope BX51, Tokyo, Japan) equipped with a camera. Myelinated profiles were digitally photographed at two different magnifications (x20 and x100). All subsequent counts were made by an independent blinded person using NIH ImageJ software (National Institutes of Health, Bethesda, USA). Global differences in the number of myelinated axons were determined by counting myelinated axons within four areas of 4004 µm^2^ in each section as described^22^. Although this approach is not the best for the evaluation of axonal calibre and myelin sheath thickness, we have chosen to use it so as to be able to perform counting analyses of myelinated axons in semithin sections and thus to determine the most remarkable effects of the cannabinoid drug WIN during the early stage of remyelination. N = 3 mice/group.

### Western blotting

The corpus callosum was homogenized in a RIPA buffer containing a mixture of protease inhibitors (Roche Applied Science, Penzberg, Germany). Twenty micrograms of protein was mixed with 5 × Laemmli buffer, then denatured for 5 min at 60 °C, separated by 10% SDS-PAGE, and finally transferred onto nitrocellulose membrane (Amersham Biosciences, Little Chalfont, UK). The blocking step was performed in 5% (w/v) non-fat dry milk in TBS with 0.1% Tween 20 (v/v) (TBS-T). The membrane was incubated in 1% (w/v) non-fat dry milk in TBS-T using the following antibodies: rabbit anti-CB_1_ receptor primary antibody (1:500; Frontier Science, Hokkaido, Japan) and rabbit anti-β-actin (1:3000; Sigma-Aldrich, Hamburg, Germany). All primary antibodies were recognized by the anti-rabbit HRP-conjugated secondary antibody (1:1500; Sigma-Aldrich, Hamburg, Germany) followed by ECL-detection (Bio-Rad, Hercules, USA). The grouping blots were cropped from different parts of the same gel of apparently irrelevant lanes and and exposed exactly the same way. Chemiluminescence was identified by Amersham HyperfilmTM ECL (GE Healthcare, Little Chalfont, UK) and then quantified with Image J software (National Institutes of Health, Bethesda, USA). N = 4mice/group.

### RNA Isolation

The corpus callosum from each of the CPZ-exposed and control mice was sonicated with a blender in RNase-free lysis buffer (Applied Biosystems, Darmstadt, Germany). Samples were kept for 1 h at 4 °C. Total RNA was obtained following a TRIzol protocol (Invitrogen Ltd., NY, USA), then digested with RNase-free DNase (Qiagen, Düsseldorf, Germany) and checked for integrity by electrophoresis (Bioanalyzer, Agilent Technologies, Santa Clara, USA). N = 3mice/group.

### Quantitative RT-PCR

cDNA was synthesized from 1 μg RNA using a High Capacity RNA-to-cDNA kit (Applied Biosystems, Darmstadt, Germany). mRNA expression was then measured by quantitative RT-PCR using CXF96TM Real-Time PCR (Bio-Rad, Hercules, USA). GAPDH mRNA was used as an endogenous control. TaqMan gene expression assays for mouse *Plp1, Pdgfra, Aif1, Gfap* and *Cntnap1* cDNAs were obtained from validated and predesigned Assays-on-Demand (Applied Biosystems, Darmstadt, Germany) and used in real time PCR amplifications to detect the expression of the genes. The reactions were performed in triplicate using 2 μl of cDNA in a 10 μl volume. The mRNA expression for each sample was determined using the comparative cycle threshold (Ct) method in accordance with the manufacturer’s instructions (Applied Biosystems, Darmstadt, Germany). The quantification of cDNAs based on 2−ΔΔCt method was performed relative to a “calibrator” control sample.

### Statistical analysis

Statistical significance was evaluated by Two-way ANOVA and the Bonferroni post hoc test when applicable. Significance was set at p < 0.05. Data are shown as mean ± SEM in figures and text if not otherwise stated. Data were analyzed using Statistica (StatSoft Software, Tulsa, USA).

### Ethical approval

All procedures were approved by the Göttingen University Institutional Animal Care and Use Committee and were in accordance with NIH guidelines for the use of animals in research and the European Communities Council Directive (2010/63/EU).

## Results

### Cuprizone feeding reduced body weight but did not alter contextual and tone fear conditioning

**Figure 2:**
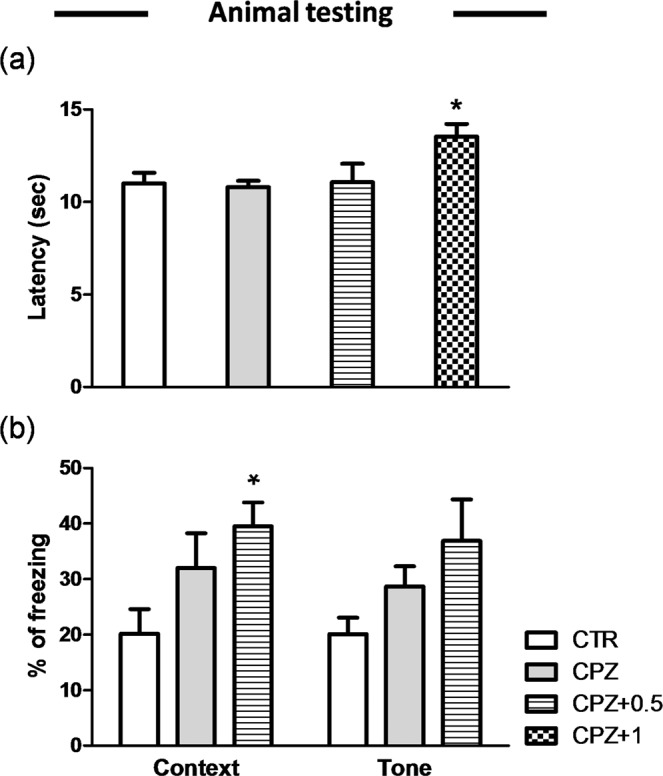
Nociception and fear response. (**a**) The daily use of WIN at 1 mg/Kg increased the time spent on the hotplate when compared to the control group (p < 0.05). (**b**) The evaluation of fear response revealed that following 3 weeks of CPZ diet in combination with 0.5 mg/Kg of WIN there was an increase of the freezing behavior during a contextual memory test performed 24 h after the training session when compared to controls (p < 0.05) while no differences were found when animals were fed with CPZ alone. Data are expressed as mean ± SEM. An * indicates significant differences between CPZ-fed groups and their respective control group. N = 15, n = 10; respectively. Control, animals fed with a standard diet and treated with phosphate buffered saline; CPZ, cuprizone-fed animals; WIN, WIN-55,212-2.

The weight of the mice was registered weekly throughout the experimental period and measured in grams. Following one week of CPZ diet, body weight was significantly lower than the control group (22.72 ± 3.20, n = 10, in CPZ alone vs. 24.01 ± 3.16, n = 10, in controls) (p < 0.05), which returned to control levels one week later (Fig. S1). In contrast, WIN-treated animals did not have significant loss of weight in comparison to controls throughout the experiment (data not shown). The daily use of WIN did not alter nociception when the drug was administered at 0.5 mg/Kg however, 1 mg/Kg of WIN increased the time spent on the hotplate (13.53 ± 0.68, n = 15, in CPZ + 1 vs. 11 ± 0.57, n = 15, in controls) (p < 0.05). Thus, we did not take in consideration the CPZ group treated with 1 mg/Kg for fear conditioning analysis. We assessed mouse behavior for fear response by registering the number of seconds spent freezing in the chamber. The evaluation of fear response revealed that following 3 weeks of CPZ diet in combination with 0.5 mg/Kg of WIN there was an increase of the freezing behavior during a contextual memory test performed 24 h after the training session when compared to controls (42.00 ± 4.30, n = 10, in CPZ + 0.5 vs. 21.50 ± 4.40, n = 10, in controls) (p < 0.05) while no differences were found when animals were fed with CPZ alone (Fig. 2). Conversely, the initial freezing response to the context and tone presentation, 24 h after conditioning, was similar for all groups at 6 and 12 weeks (data not shown).

### CNS myelination is impaired when the drug is administered at 1 mg/Kg while 0.5 mg/Kg favours neuroprotection and myelin repair

**Figure 3:**
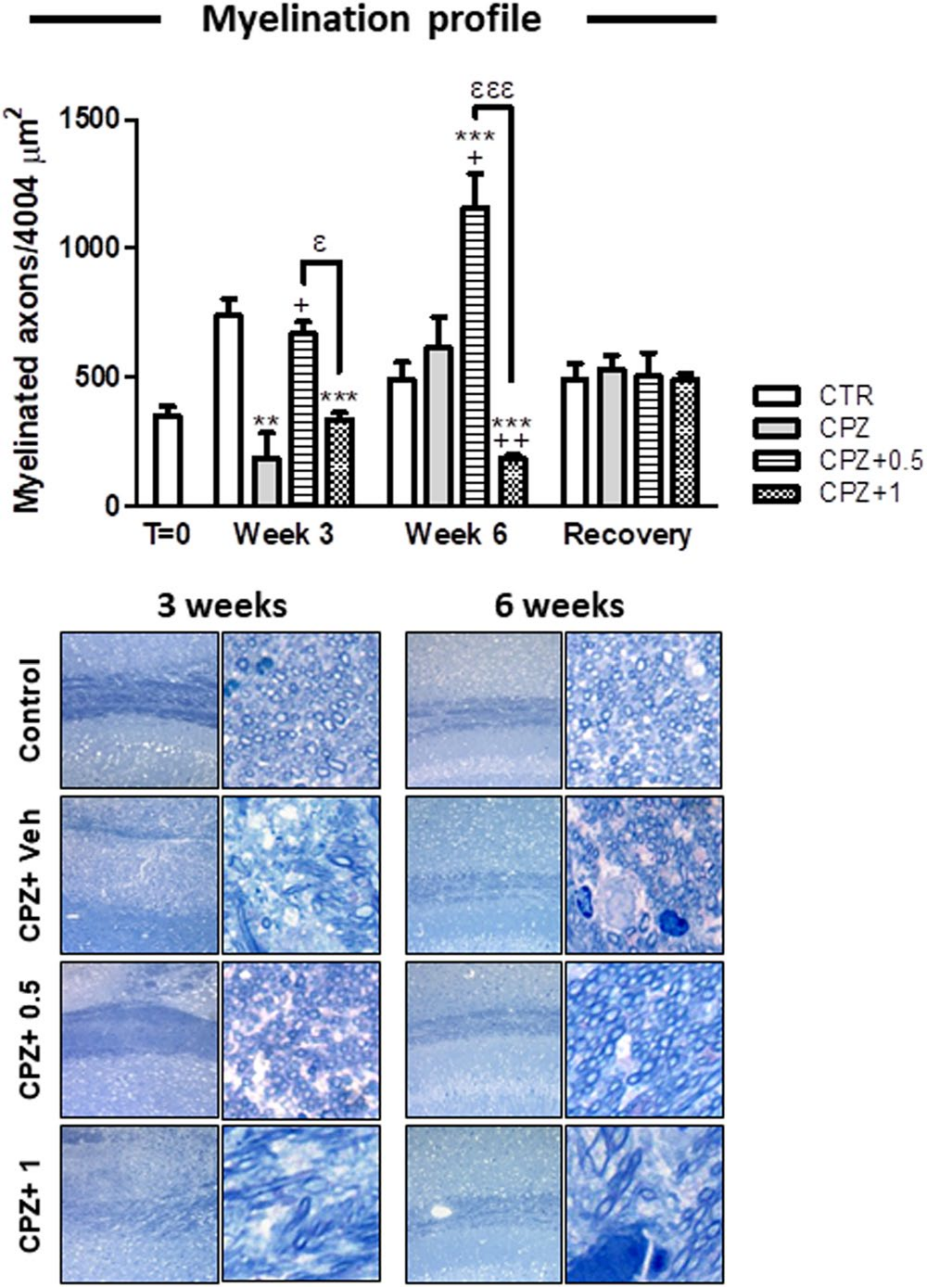
The corpus callosum myelination. Labeled structures were digitally photographed using x20 (left column) and x100 (right column) magnification. We reported a clear demyelination after three weeks of CPZ alone when compared to controls (p < 0.01) while CPZ-fed mice subjected to 0.5 mg/Kg of WIN displayed no significant differences in contrast to controls. However, the administration of WIN at 1 mg/Kg in CPZ-fed mice reduced the number of myelinated axons when compared to either controls (p < 0.001) or CPZ-fed animals treated with 0.5 mg/Kg of WIN (p < 0.01). Mice exposed to CPZ alone for six weeks displayed similar profiles of myelinated fibers than controls while animals exposed to CPZ and treated with 0.5 mg/Kg of the drug showed more myelinated fibers than either controls (p < 0.001), CPZ alone (p < 0.05) or CPZ-fed mice subjected to 1 mg/Kg of WIN (p < 0.001). In contrast, CPZ-fed animals treated with 1 mg/Kg showed lower counts of myelinated axons than either controls (p < 0.001), CPZ alone (p < 0.01) or CPZ-fed mice treated with 0.5 mg/Kg (p < 0.001). Data are expressed as mean ± SEM. An * indicates significant differences between CPZ-fed groups and their respective control group. Comparisons between the group exposed to CPZ alone and those animals treated simultaneously with both CPZ and WIN are indicated by an+. An underlined Ɛ indicated comparisons between all WIN treated groups. One, two or three symbols indicate p < 0.05; p < 0.01; p < 0.001, respectively. N = 3. Control, animals fed with a standard diet and treated with phosphate buffered saline; CPZ, cuprizone-fed animals; WIN, WIN-55,212-2.

Here we evaluated the consequences of the administration of the cannabinoid agonist WIN at specific time points of the CPZ model in order to elucidate the contribution of this drug to the CNS myelination. The corpus callosum was extensively analyzed by methylene blue AZUR II staining and representative pictures are presented in Fig. 3.

Counting analysis of myelinated fibers indicated a clear demyelination following three weeks of treatment with CPZ alone when compared to controls (182.50 ± 97.66, n = 3, in CPZ alone vs. 736.50 ± 63.64, n = 3, in controls) (p < 0.01) while co-administration of CPZ and 0.5 mg/Kg of WIN revealed no significant differences in comparison to controls but significantly more myelinated axons than the group fed with CPZ alone (666.00 ± 50.09, n = 3, in CPZ + 0.5 vs. 182.50 ± 97.66, n = 3, in CPZ alone) (p < 0.05) (Fig. 3). During the acute demyelination (week 3), the administration of WIN at 1 mg/Kg in CPZ-fed mice impaired the myelination process when compared to either controls (334.50 ± 26.24, n = 3, in CPZ + 1 vs. 736.50 ± 63.64, n = 3, in controls) (p < 0.001) or CPZ-fed animals treated with 0.5 mg/Kg of WIN (334.50 ± 26.24, n = 3, in CPZ + 1 vs. 666.00 ± 50.09, n = 3, in CPZ + 0.5) (p < 0.01) (Fig. 3).

Mice exposed to the neurotoxicant alone for six weeks displayed similar number of myelinated fibers than the controls (Fig. 3). At six weeks of CPZ exposure, mice treated with 0.5 mg/Kg of WIN showed greater remyelinating potential than the remaining groups (488.50 ± 67.88, n = 3, in controls; 611.00 ± 119.44, n = 3, in CPZ alone; 183.00 ± 11.38, n = 3, in CPZ + 1 vs. 1153.50 ± 132.82, n = 3, in CPZ + 0.5) (p < 0.001; p < 0.05; p < 0.001, respectively) (Fig. 3). This data is in line with our previous findings, which in turn underpin a plausible neuroprotective effect mediated by the drug when it is administered at 0.5 mg/Kg. During the early remyelination stage (week 6), treatment with 1 mg/Kg of WIN limited myelin repair capacity when compared to either controls (183.00 ± 11.38, n = 3, in CPZ + 1 vs. 488.50 ± 67.88, n = 3, in controls) (p < 0.001), CPZ alone (183.00 ± 11.38, n = 3, in CPZ + 1 vs. 611.00 ± 119.44, n = 3, in CPZ alone) (p < 0.01) or CPZ-fed mice treated with 0.5 mg/Kg (183.00 ± 11.38, n = 3, in CPZ + 1 vs. 1153.50 ± 132.82, n = 3, in CPZ + 0.5) (p < 0.001) (Fig. 3).

Finally, the myelination of corpus callosum was completely restored after arecovery period of six weeks withdrawal CPZ as shown in Fig. 3.

### Long-term administration of WIN-55,212-2 reduced the content of CB_1_ receptor

**Figure 4:**
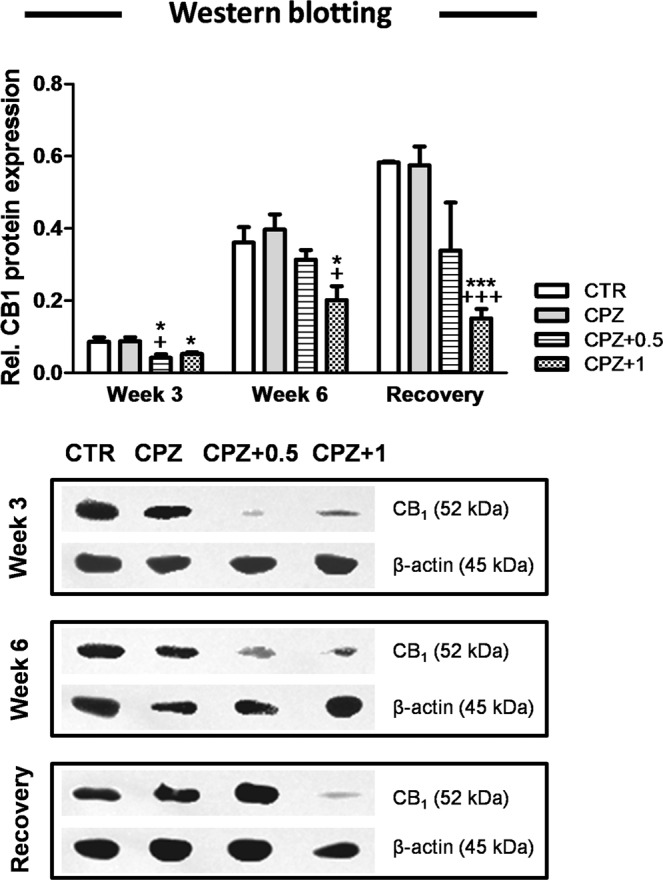
The cannabinoid CB_1_ protein. At the third week, the CB_1_ protein levels were significantly higher in controls when compared to CPZ-fed mice treated with either 0.5 or 1 mg/Kg of WIN (p < 0.05). During the acute phase of demyelination (week 3), animals exposed to CPZ alone showed an increase in the CB_1_ protein when compared to CPZ-fed mice subjected to 0.5 mg/Kg of WIN (p < 0.05). Moreover, animals fed with CPZ in combination with 1 mg/Kg of WIN exhibited less CB_1_ protein than either controls or CPZ alone at six (p < 0.05) and twelve weeks (p < 0.001). Data are expressed as mean ± SEM. An * indicates significant differences between CPZ-fed groups and their respective control group. Comparisons between the group exposed to CPZ alone and those animals treated simultaneously with both CPZ and WIN are indicated by an + . Anunderlined Ɛ indicated comparisons between all WIN treated groups. One or three symbols indicate p < 0.05; p < 0.001, respectively. N = 4. Control, animals fed with standard diet and treated with phosphate buffered saline (Veh); CPZ, cuprizone-fed animals; WIN, WIN-55,212-2.

We examined the amount of protein for CB_1_ receptor in the corpus callosum homogenates collected from all experimental groups by use of quantitative Western blotting (Fig. 4).

In the third week, when maximum demyelination occurs, the content of CB_1_ protein was significantly higher in controls than in CPZ-fed mice treated with either 0.5 or 1 mg/Kg WIN following normalization to β-actin (0.04 ± 0.01, n = 4, in CPZ + 0.5; 0.05 ± 0.00, n = 4, in CPZ + 1 vs. 0.08 ± 0.01, n = 4, in controls) (p < 0.05) (Fig. 4). During the acute phase of demyelination (week 3), animals exposed to CPZ alone were found to have larger amounts of CB_1_ protein than CPZ-fed mice subjected to 0.5 mg/Kg of WIN (0.09 ± 0.01, n = 4, in CPZ alone vs. 0.04 ± 0.01, n = 4, in CPZ + 0.5) (p < 0.05) (Fig. 4).

Animals fed with CPZ and simultaneously treated with 1 mg/Kg of the drug showed a decrease in the CB_1_ protein content in comparison to both controls and CPZ alone at six (0.36 ± 0.04, n = 4, in controls; 0.40 ± 0.04, n = 4, in CPZ alone vs. 0.20 ± 0.04, n = 4, in CPZ + 1) (p < 0.05) (Fig. 4) and twelve weeks (0.58 ± 0.00, n = 4, in controls; 0.57 ± 0.05, n = 4, in CPZ alone vs. 0.15 ± 0.02, n = 4, in CPZ + 1) (p < 0.001) (Fig. 4).

### WIN-55,212-2 differentially deregulated gene expression of glia but did not alter axonal integrity

Quantitative RT-PCR was directed to quantify markers of inflammation (*Gfap* as a marker of reactive glia and *Aif1* as a marker of monocyte-macrophages), myelination (*Plp1* as a marker of myelinating oligodendrocyte and *Pdgfra* as a marker of oligodendrocyte precursor cells (OPC)) and axonal integrity (*Cntnap1*). These markers were accurately chosen in order to understand the pathophysiology of demyelination and also characterize the mechanisms involved in the remyelination process^10^.

**Figure 5:**
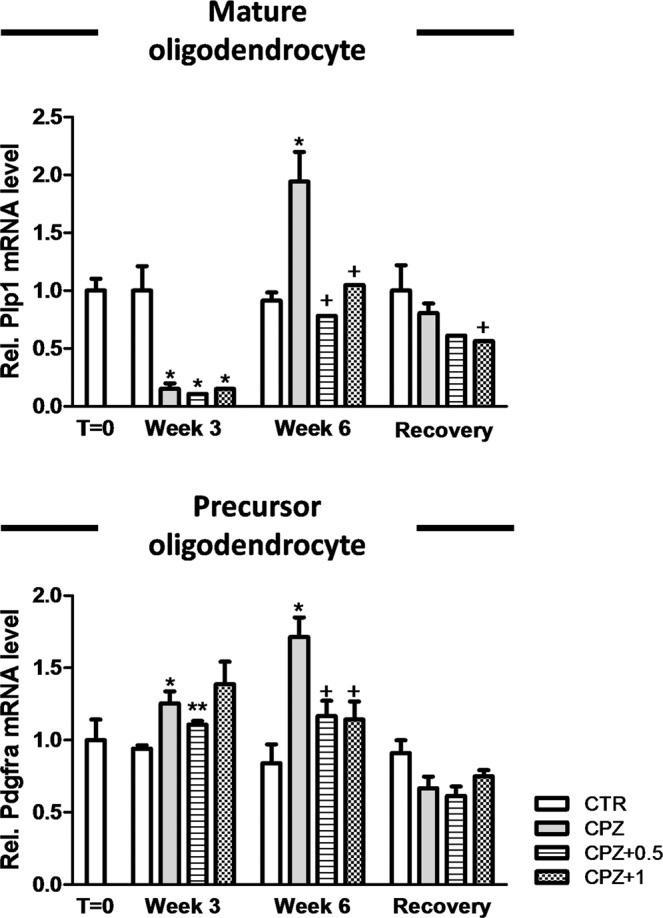
Relative mRNA expression for oligodendrocyte markers. Animals exposed for three weeks to CPZ diet and treated with either the vehicle, 0.5 or 1 mg/Kg of WIN showed lower expression of myelin/oligodendrocyte-related gene (*Plp1*) in comparison to controls (p < 0.05) while the expression of OPCs (*Pdgfra*) was higher in CPZ-fed mice subjected to either vehicle or 0.5 mg/Kg of WIN than the control group (p < 0.05; p < 0.01, respectively). At week 6, the expression of *Plp1* was significantly greater in animals fed with CPZ alone when compared to either controls, 0.5 or 1 mg/Kg of WIN (p < 0.05). During the early remyelination phase, the gene expression for *Pdgfra* increased following exposure to CPZ alone in comparison to both controls (p < 0.05) and CPZ-fed animals subjected to either 0.5 or 1 mg/Kg of WIN (p < 0.05) while no significant effects were observed in CPZ-fed animals treated with either 0.5 or 1 mg/Kg of WIN when compared to controls. 6 weeks after CPZ withdrawal, mice treated with vehicle showed an increase of *Plp1* expression in contrast to those treated with 1 mg/Kg of WIN (p < 0.05). Data are expressed as mean ± SEM. An * indicates significant differences between CPZ-fed groups and their respective control group. Comparisons between the group exposed to CPZ alone and those animals treated simultaneously with both CPZ and WIN are indicated by an + . An underlined Ɛ indicated comparisons between all WIN treated groups. One or two symbols indicate p < 0.05 and p < 0.001, respectively. N = 3. Control, animals fed with standard diet and treated with phosphate buffered saline; CPZ, cuprizone-fed animals; WIN, WIN-55,212-2; OPC, oligodendrocyte precursor cells.

In brief, animals exposed to the neurotoxicant for three weeks and treated with either the vehicle, 0.5 or 1 mg/Kg of the drug had a decrease in the myelin/oligodendrocyte-related gene (*Plp1*) in comparison to controls (0.15 ± 0.04, n = 3, in CPZ alone; 0.11 ± 0.01, n = 3, in CPZ + 0.5; 0.15 ± 0.01, n = 3, in CPZ + 1 vs. 1.00 ± 0.21, n = 3, in controls) (p < 0.05) (Fig. 5) while the expression of OPC marker (*Pdgfra*) was markedly higher in CPZ-fed mice subjected to either the vehicle or 0.5 mg/Kg of WIN than the control group (1.25 ± 0.08, n = 3, in CPZ alone; 1.11 ± 0.02, n = 3, in CPZ + 0.5 vs. 0.94 ± 0.02, n = 3, in controls) (p < 0.05; p < 0.01, respectively) (Fig. 5).

**Figure 6:**
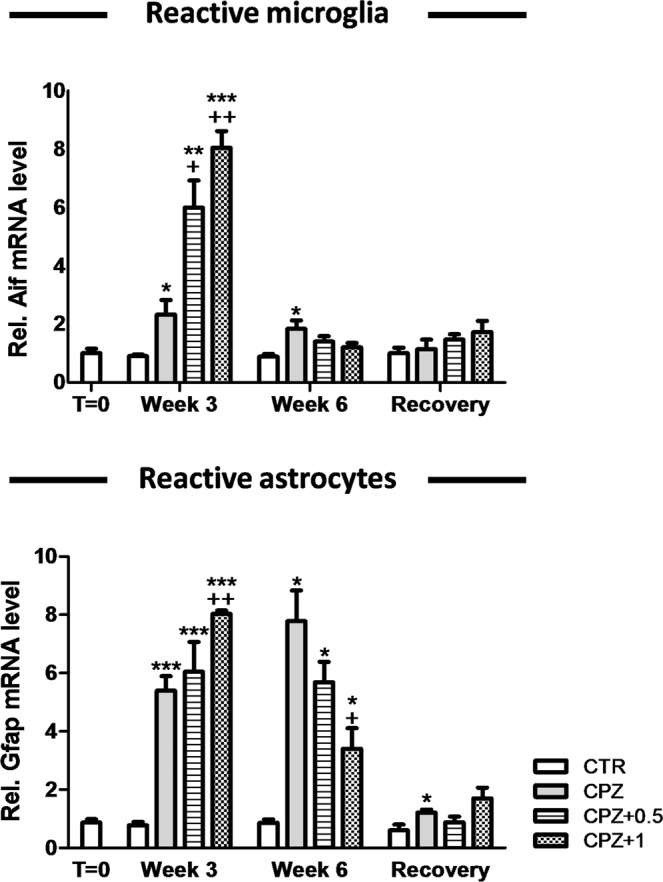
Relative mRNA expression for inflammatory markers. We reported a significant increase in reactive microglia (*Aif1*) and astrogliosis (*Gfap*) following three weeks with a CPZ diet when it was combined with either the vehicle, 0.5 or 1 mg/Kg of WIN (for *Aif1*, p < 0.05; p < 0.01; p < 0.001, respectively while for *Gfap*, p < 0.001). At week 3, the administration of both CPZ and 0.5 mg/Kg of WIN increased the expression of *Aif1* when compared to CPZ alone (p < 0.05). In addition, we measured an increase in both *Gfap* and *Aif1* gene expression following 3 weeks with CPZ supplemented diet and 1 mg/Kg of WIN when compared to controls (p < 0.001) or CPZ alone (p < 0.01) while no effects were found in comparison to 0.5 mg/Kg of WIN. After six weeks of CPZ administration, we found a greater expression of *Aif1* in CPZ-fed animals treated with the vehicle (p < 0.05) and also a higher transcription rate for *Gfap* in those animals fed with CPZ and simultaneously treated with either vehicle, 0.5 or 1 mg/Kg of WIN (p < 0.05) than their controls. During the early remyelination phase, animals fed with CPZ and treated with 1 mg/Kg of WIN had less *Gfap* expression than those fed with CPZ alone (p < 0.05). Six weeks after CPZ withdrawal, mice treated with vehicle underwent an increase in *Gfap* expression when compared to controls (p < 0.05). Data are expressed as mean ± SEM. An * indicates significant differences between CPZ-fed groups and their respective control group. Comparisons between the group exposed to CPZ alone and those animals treated simultaneously with both CPZ and WIN are indicated by an +. Anunderlined Ɛ indicated comparisons between all WIN treated groups. One, two or three symbols indicate p < 0.05, p < 0.01, and p < 0.001, respectively. N = 3. Control, animals fed with a standard diet and treated with phosphate buffered saline (Veh); CPZ, cuprizone-fed animals; WIN, WIN-55,212-2.

During the acute demyelination (week 3), reactive microglia (*Aif1*) was reported in CPZ-fed animals treated with either the vehicle, 0.5 or 1 mg/Kg of WIN (2.33 ± 0.49, n = 3, in CPZ alone; 5.99 ± 0.94, n = 3, in CPZ + 0.5; 8.04 ± 0.57, n = 3, in CPZ + 1 vs. 0.91 ± 0.05, n = 3, in controls) (p < 0.05; p < 0.01; p < 0.001, respectively) when compared to controls (Fig. 6). The marker *Gfap* was used to assess astrocyte reactivity in our model. By the third week, *Gfap* was up-regulated in CPZ-fed mice treated with the vehicle, 0.5 and 1 mg/Kg of WIN when compared to controls (5.39 ± 0.49, n = 3, in CPZ alone; 6.05 ± 1.02, n = 3, in CPZ + 0.5; 8.03 ± 0.12, n = 3, in CPZ + 1 vs. 0.77 ± 0.12, n = 3, in controls) (p < 0.001) (Fig. 6). The administration of CPZ for three weeks along with 0.5 mg/Kg of WIN increased *Aif1* expression when compared to CPZ alone (5.99 ± 0.94, n = 3, in CPZ + 0.5 vs. 2.33 ± 0.49, n = 3, in CPZ alone) (p < 0.05) (Fig. 6). The inflammatory reaction observed following three weeks of CPZ feeding was remarkably stronger in those animals subjected to the pharmacological action of 1 mg/Kg of the drug (Fig. 6). Indeed, CPZ-fed animals exposed to this dosage of WIN showed an increase in both *Gfap* (8.03 ± 0.12, n = 3, in CPZ + 1 vs. either 0.77 ± 0.12, n = 3, in controls or 5.39 ± 0.49, n = 3, in CPZ alone) and *Aif1* (8.04 ± 0.57, n = 3, in CPZ + 1 vs. either 0.91 ± 0.05, n = 3, in controls or 2.33 ± 0.49, n = 3, in CPZ alone) expression when compared to controls (p < 0.001) or CPZ alone (p < 0.01) while, in contrast, no effects were found to CPZ-fed mice treated with 0.5 mg/Kg (Fig. 6).

At week 6, when mice were still undergoing exposure to CPZ, the expression of *Plp1* was significantly higher in those animals treated only with the neurotoxicant than either controls or WIN-treated groups (0.91 ± 0.07, n = 3, in controls; 0.78 ± 0.08, n = 3, in CPZ + 0.5; 1.05 ± 0.18, n = 3, in CPZ + 1 vs. 1.94 ± 0.25, n = 3, in CPZ alone) (p < 0.05) (Fig. 5). However, animals fed with CPZ and treated with either 0.5 or 1 mg/Kg of the drug displayed similar expression for *Plp1* than the controls (Fig. 5). During the early remyelination stage, levels of *Pdgfra* were significantly greater in CPZ-fed animals treated with the vehicle than controls (0.84 ± 0.13, n = 3, in controls vs. 1.71 ± 0.13, n = 3, in CPZ alone) (p < 0.05) and CPZ-fed animals exposed to either 0.5 or 1 mg/kg of WIN (1.17 ± 0.11, n = 3, in CPZ + 0.5; 1.14 ± 0.12, n = 3, in CPZ + 1 vs. 1.71 ± 0.13, n = 3, in CPZ alone) (p < 0.05) while no notable differences were detected in CPZ-fed animals subjected to the drug (Fig. 5). After six weeks of CPZ feeding, reactive microglia and astrogliosis were actively present (Fig. 6). CPZ-fed animals treated with the vehicle showed an up-regulation of *Aif1* when compared to controls (1.84 ± 0.29, n = 3, in CPZ alone vs. 0.88 ± 0.09, n = 3, in controls) (p < 0.05) and there was also *Gfap* expression in CPZ-fed mice treated with either the vehicle, 0.5 or 1 mg/Kg of WIN (7.78 ± 1.05, n = 3, in CPZ alone; 5.69 ± 0.69, n = 3, in CPZ + 0.5; 3.40 ± 0.70, n = 3, in CPZ + 1 vs. 0.86 ± 0.11, n = 3, in controls) (p < 0.05) in contrast to the control group (Fig. 6). After six weeks of CPZ feeding, animals subjected to 1 mg/Kg of the drug showed a lower number of *Gfap* transcripts than animals fed with CPZ alone (3.40 ± 0.70, n = 3, in CPZ + 1 vs. 7.78 ± 1.05, n = 3, in CPZ alone) (p < 0.05) (Fig. 6).

After CPZ feeding, the animals were allowed to recover on a regular chow diet for 6 weeks (the recovery group). All gene expression markers presented here returned to basal conditions but were not reestablished when mice were fed with CPZ alone. In fact, CPZ-fed mice subjected to daily vehicle administration showed a robust increment in *Plp1* expression when compared to CPZ-fed mice treated with 1 mg/kg of the drug (0.80 ± 0.08, n = 3, in CPZ alone vs. 0.56 ± 0.04, n = 3, in CPZ + 1) (p < 0.05) (Fig. 5) while *Gfap* was over-expressed in comparison to controls (1.21 ± 0.11, n = 3, in CPZ alone vs. 0.60 ± 0.20, n = 3, in controls) (p < 0.05) (Fig. 6). Finally, gene expression analysis for *Cntnap1* did not reveal significant differences between groups (Fig. S2) and so we can assume that the administration of the neurotoxicant CPZ in combination with the vehicle or WIN did not alter the axonal integrity.

## Discussion

It is widely accepted that the long-term administration of cannabinoid agonists produces tolerance to cannabinoid-related effects^23^. Cellular adaptations to chronic cannabinoid drug administration include a decrease in CB_1_ levels and also a desensitization of CB_1_-mediated G protein activation^6,24^. In line with these findings, we reported that daily administration of WIN reduced the global amount of CB_1_ protein in the corpus callosum. However, we observed no notable effects on the content of CB_1_ protein in those animals exposed to CPZ alone, unlike the findings of some earlier studies^25,26^. Possible explanations for these discrepancies are the species used (rats or mice) and differences in the analytical methods applied and sampling times. In this work, we demonstrated that the CPZ *per se* did not impair fear conditioning to context and tone presentation as described^27,28^. The acute administration of the cannabinoid WIN severely impaired contextual conditioning but did not modify the conditioning to a tone^29^. In contrast, systemic administration of 1 mg/Kg of WIN produced changes in nociceptive neurotransmission and led to the development of antinociceptive reactions^30^ which did not occur when the drug was administered at 0.5 mg/Kg.

By three weeks, there was an evident loss of myelin in CPZ-fed animals treated with vehicle, which in turn was associated with a dramatic down-regulation of *Plp1* expression as has previously been demonstrated^31^. Conversely, histological analysis of the demyelinated area revealed that CNS myelination was slightly compromised when 0.5 mg/Kg of WIN was administered. Similarly, a previous study revealed that the cannabinoid WIN at 0.5 mg/Kg confers neuroprotection against demyelination while the administration of the drug at 1 mg/Kg aggravates the process^5^. Despite this, it is widely accepted that the cannabinoid receptors modulate the severity of demyelination in distinct experimental animal models^5,32^. The administration of CPZ for three weeks led to a pronounced inflammatory reaction by activating the recruitment of microglia and astroglial cells in the demyelinated area^10,33^. In addition, we demonstrated that the cannabinoid drug potentiates the expression of markers for microglia and astrocytes in a dose-dependent manner. The agonist WIN could interact with the cannabinoid receptors (CB_1_ or CB_2_) or with noncannabinoid receptors expressed on astrocytes and microglial cells favouring their activation^34–36^. Therefore, the activation of both astrocyte and microglial cells by the agonist WIN warrants further investigation. The overall differences observed during demyelination on drug treatment could be attributable in part to a specific deregulation of astrocyte reactivity, since recent findings suggest that astroglial cells are actively involved during oligodendrocyte degeneration, controlling local CNS inflammation^37^. CPZ-fed animals exposed daily to the vehicle displayed lower transcription rates for *Plp1* (marker for mature oligodendrocytes) while the expression of *Pdgfra* (marker for OPCs) was markedly higher^10,33^. Nevertheless, when the drug was administered at 1 mg/Kg, this increase was not observed, showing that the proliferation of OPCs could be seriously compromised in these animals. During the remyelination stage (week 6), CPZ-fed animals treated with the vehicle showed considerable spontaneous remyelination as indicated by the greater number of myelinated axons and also an increase in both *Plp1* and *Pdgfra* expression^10^. Conversely, the administration of the drug during the remyelination phase (week 6) had no notable effects on the expression of these oligodendrocyte markers but it had significant consequences on the number of myelinated axons. It is therefore necessary for further investigation to address the use of a set of oligodendrocyte markers in order to characterize histologically and transcriptionally the oligodendrocyte lineage. When CPZ is orally administered, animals displayed a declined activity of copper-zinc superoxide dismutase^38^ which in turn, could be counteracted by use of cannabinoid drugs as a protective mechanism against reactive oxygen metabolites damage^39^. Despite this, several independent groups have demonstrated that the activation of CB_1_ receptors in outer mitochondrial membranes regulates respiratory chain complexes and thus, mitochondrial biogenesis (for review see^40^). Thus, it might be speculate that the drug at 0.5 mg/kg dosage could possess a therapeutic effect probably by protecting neurons against CPZ-induced neurotoxicity through the stimulation of copper-zinc superoxide dismutase enzyme^39^ or by promoting the differentiation of oligodendrocytes^5^. On the other hand, 1 mg/kg of the drug had a deleterious effect, presumably related to an inhibitory effect of G_i_/G_o_-proteins expressed in neurons as our group postulated on previously^5^. By week 6, microglia and astrocytes remained active in the corpus callosum of animals exposed to CPZ alone, in part, to clear myelin debris^10^ and also to provide metabolic support to the neurons^41^. Nevertheless, the expression of *Aif1* was attenuated when the drug was administered during the remyelination phase. Defaux *et al*.^42^ demonstrated that wh en microglia reactivity was chemically repressed, animals displayed better remyelination potential with no effect in the event of a demyelinating insult. However, some authors described the opposite^9,37^. Therefore, this issue warrants further investigation. In addition, it is well known that astrocytes can release certain molecules potentiating remyelination or its failure in a neurodegenerative pathologic state^43^. In the recovery group, the corpus callosum myelination was apparently normal^5,10,44^. However, astrocytes remained functionally active in CPZ-fed animals subjected to the vehicle. In addition, no obvious differences in axonal integrity were found between the groups throughout the experimental period^10,44,45^.

In summary, the data reported here highlights the impact of the cannabinoid WIN on gene expression of certain markers for astrocytes, microglia and oligodendrocytes in the CPZ animal model of MS. Histological analysis of the corpus callosum revealed that myelination was slightly compromised when 0.5 mg/Kg of WIN was administered due to the fact that this dosage protected neurons against CPZ-induced neurotoxicity probably by enhancing copper-zinc superoxide dismutase activity^39^ or by promoting oligodendrocyte differentiation^5^. In contrast, high dosage of WIN had a deleterious effect, presumably related to a reduction in the L-channel opening time and calcium influx into the neurons, which in turn could be attributable to an inhibitory effect of G_i_/G_o_-proteins^46^. Similarly, different groups hav e found that the enhancement of endocannabinoid signaling promotes neuroprotection in Theiler’s virus-induced demyelinating disease^47^ as well as in experimental allergic encephalomyelitis^48^. From a translational science and a clinical point of view, accumulating evidence suggests that humans need a dosage by weight about 7 and 12 times lower than lab rodents for an equivalent cannabinoid effect^49^. Subsequently, we can speculate that the drug would possess a therapeutic effect when it is administered at doses between 0.041–0.071 mg/Kg in patients diagnosed with MS. We conclude that the therapeutic use of WIN warrant further investigation and should be accompanied by histological evidences.

## Supplementary information

Supplementary information.

Supplementary information2.
