# Supplementary material for: Dose-dependent effect of cannabinoid WIN-55,212-2 on myelin repair following a demyelinating insult
Source: Sci Rep. 2020 Jan 17;10:590. doi: 10.1038/s41598-019-57290-1 (PMC6969154; doi:10.1038/s41598-019-57290-1)
Supplement: Supplementary file 1 — Supplementary information. [file 41598_2019_57290_MOESM1_ESM.pdf]

Article in Scientific Reports

Dose-dependent effect of cannabinoid WIN-55,212-2 on myelin repair following a demyelinating insult

J. Tomas-Roig, HY. Agbemenyah, N. Celarain, E. Quintana, Ll. Ramió-Torrentà and U. Havemann-Reinecke

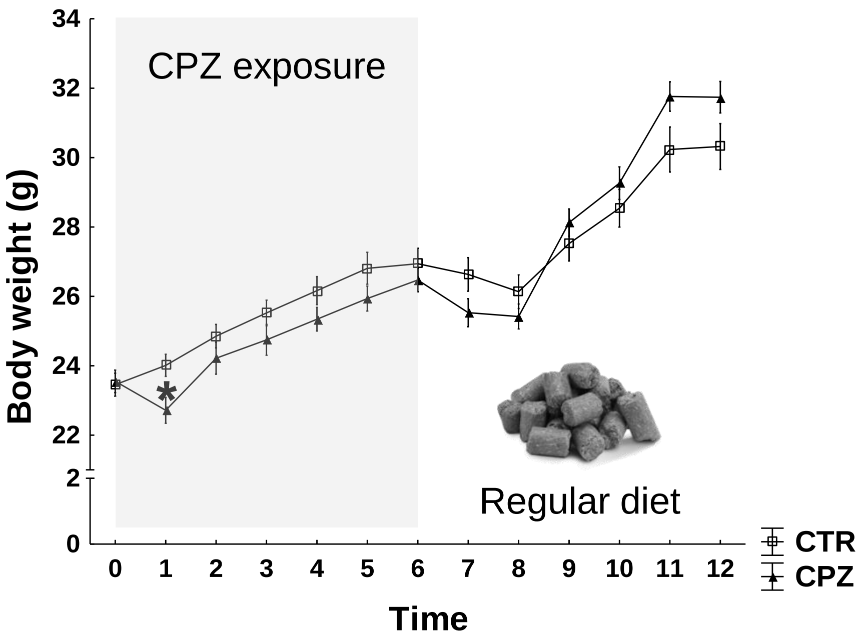

Fig. S1. Body weight throughout the experimental period. Following one week of cuprizone supplemented diet, mice body weight was lower than controls ($p<0.05$) while it was restored to control levels one week later. Administration of CPZ and WIN simultaneously did not reveal significant effects on body weight in contrast to the control group (data not shown). Data are expressed as mean ± SEM. P value was set at *$p<0.05$. N= 10. Control, animals fed with a standard diet and treated with phosphate buffered saline (Veh); CPZ, cuprizone-fed animals.
