# Supplementary material for: Dose-dependent effect of cannabinoid WIN-55,212-2 on myelin repair following a demyelinating insult
Source: Sci Rep. 2020 Jan 17;10:590. doi: 10.1038/s41598-019-57290-1 (PMC6969154; doi:10.1038/s41598-019-57290-1)
Supplement: Supplementary file 2 — Supplementary information2. [file 41598_2019_57290_MOESM2_ESM.pdf]

Article in Scientific Reports

Dose-dependent effect of cannabinoid WIN-55,212-2 on myelin repair following a demyelinating insult

J. Tomas-Roig, HY. Agbemenyah, N. Celarain, E. Quintana, Ll. Ramió-Torrentà and U. Havemann-Reinecke

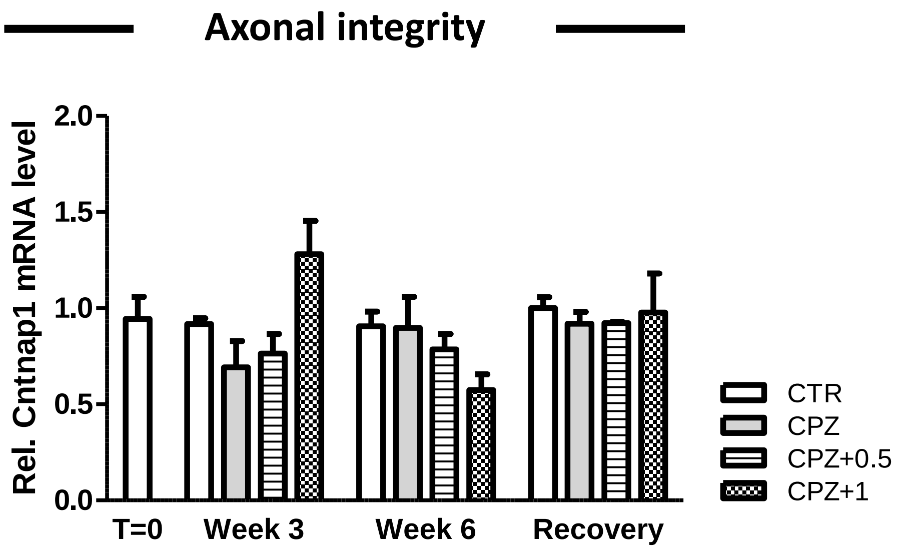

Fig. S2. Relative mRNA expression for a marker of axonal integrity. We did not find significant differences in axonal integrity throughout the experimental period as indicated by the expression of the *Cntnap1* marker. Data are expressed as mean ± SEM. N= 3. Control, animals fed with standard diet and treated with phosphate buffered saline (Veh); CPZ, cuprizone-fed animals; WIN, WIN-55,212-2.
